# Antibacterial and Antibiofilm Activity of Myrtenol against *Staphylococcus aureus*

**DOI:** 10.3390/ph13060133

**Published:** 2020-06-25

**Authors:** Laísa Cordeiro, Pedro Figueiredo, Helivaldo Souza, Aleson Sousa, Francisco Andrade-Júnior, José Barbosa-Filho, Edeltrudes Lima

**Affiliations:** 1 Department of Pharmaceutical Science, Health Sciences Center, Federal University of Paraíba, João Pessoa 58033-455, Paraíba, Brazil; pedrotrfigueiredo@gmail.com (P.F.); aleson_155@hotmail.com (A.S.); juniorfarmacia.ufcg@outlook.com (F.A.-J.); jbarbosa@ltf.ufpb.br (J.B.-F.); edelolima@yahoo.com.br (E.L.); 2 Chemistry Department, Exact and Natural Sciences Center, Federal University of Paraíba, João Pessoa 58033-455, Brazil; helivaldog3@gmail.com

**Keywords:** myrtenol, *Staphylococcus aureus*, antibacterial, molecular docking, antibiofilm, checkerboard method

## Abstract

The increase in *Staphylococcus aureus* resistance to conventional antibacterials and persistent infections related to biofilms, as well as the low availability of new antibacterial drugs, has made the development of new therapeutic alternatives necessary. Medicinal plants are one of the main sources of bioactive molecules and myrtenol is a natural product with several biological activities, although its antimicrobial activity is little explored. Based on this, the objective of this study was to evaluate the antibacterial activity of myrtenol against *S. aureus*, determining the minimum inhibitory and bactericidal concentrations (MIC and MBC), investigating the possible molecular target through the analysis of molecular docking. It also aimed to evaluate the effect of its combination with antibacterial drugs and its activity against *S. aureus* biofilms, in addition to performing an in silico analysis of its pharmacokinetic parameters. Myrtenol showed MIC and MBC of 128 µg/mL (bactericidal action) and probably acts by interfering with the synthesis of the bacterial cell wall. The effects of the association with antibacterials demonstrate favorable results. Myrtenol has remarkable antibiofilm activity and in silico results indicate a good pharmacokinetic profile, which make myrtenol a potential drug candidate for the treatment of infections caused by *S. aureus*.

## 1. Introduction

*Staphylococcus aureus* is an important human pathogen that causes community and nosocomial infections, associated with high morbidity and mortality, resulting in considerable costs for health systems [1]. This species is naturally susceptible to virtually all antibiotics that have ever been developed. However, it also demonstrates a unique ability to quickly develop an antibiotic resistance mechanism. The finding of *S. aureus* resistance to commonly used antibiotics, especially beta-lactams, the emergence of strains resistant to methicillin and vancomycin, and the presence of mobile genetic elements involved in the transmission of multiple antibiotic resistance genes, are complicating factors for the treatment of these infections [2]. Currently, finding new drugs to treat infections caused by *S. aureus* resistant to methicillin and vancomycin is considered a high priority [3].

One of the reasons that makes infections caused by *S. aureus* more delicate in its prognosis and treatment is the ability of this microorganism to form biofilms [4]. These structures represent a clinical challenge, as they are highly resistant to antimicrobial therapies and host defenses, generally occurring in locations that are not easily accessible for treatment and which contribute to persistent infections [5]. An aggravating factor in the fight against these infections is that some drugs, in sub-inhibitory concentrations, lead to an increase in the formation of biofilm, hindering the course of infection and treatment [6].

There is a need to develop new therapeutic alternatives, due to the existence and constant evolution of resistant microorganisms and phenotypes, the emergence of new diseases, the toxicity of some of the current antimicrobials and the scarce existence of products with antibiofilm activity. In this scenario, medicinal plants are one of the largest sources of bioactive molecules for antibacterial and antifungal purposes. One of the main advantages of studying molecules of plant origin, as observed, is the reduced number of possible toxic effects caused by such substances and the fact that they present activity, even against strains resistant to conventional antimicrobials [7]. In addition, products of natural origin can show considerable efficacy against biofilms formed by different types of bacteria [8].

As part of the context of natural products, myrtenol is a phytoconstituent present in *Myrtus communis* L. (Myrtaceae) essential oil [9], which ethnopharmacological studies revealed to be used in folk medicine for several purposes, including antimicrobial [10]. Although anxiolytic [11], anti-inflammatory [12] and gastroprotective [13] activity of isolated myrtenol has been reported; its antibacterial and antibiofilm potential is still poorly described [14]. Given these facts, this study aimed to investigate the myrtenol activity against *S. aureus*, also analyzing its effect in combination with conventional antibacterial drugs. The objective was also to evaluate the action of myrtenol on biofilms formed by *S. aureus* and to verify its pharmacokinetic parameters in silico, in order to contribute to the elucidation of its biological activities and its potential as a candidate for antibacterial drugs.

## 2. Results and Discussion

### 2.1. Determination of Minimum Inhibitory Concentration (MIC) and Minimum Bactericidal Concentration (MBC) of Myrtenol against S. aureus

To assess the antibacterial activity of myrtenol on *S. aureus*, the minimum inhibitory concentration (MIC) and minimum bactericidal concentration (MBC) were determined. The values are shown in Table 1. The MIC of myrtenol was 128 µg/mL for all *S. aureus* strains used in this study and the MBC was also 128 µg/mL for all strains tested (Table 1).

The MIC of myrtenol against *S. aureus* indicate the antibacterial activity of myrtenol, which proved to be more potent than the reports found in the literature using the isolated compound against other strains. Al-Mariri et al. [15] investigated the antibacterial activity of myrtenol isolated from the *Myrtus communis* L. essential oil and observed antibacterial activity against different Gram-negative species, with MIC ranging from 25 to 50 µL/mL. İşcan [16] evaluated the antimicrobial activity of constituents commonly found in essential oils, including myrtenol, and identified antibacterial activity ranging from 1 to 4 mg/mL for Gram-positive and negative bacteria. The MIC determined against *S. aureus* (ATCC 43300) was 2 mg/mL. Selvaraj et al. [14] determined the effect of myrtenol isolated against Methicillin-resistant *Staphylococcus aureus* (MRSA) ATCC-33591 strain and clinical isolates, identifying MIC of 600 µg/mL.

The MIC/MBC ratio was 1:1. A drug is considered to exhibit bactericidal activity against a particular isolate when the MBC/MIC ratio is ≤4 [17,18]. In this way, myrtenol exhibits antibacterial activity against *S. aureus*, acting in a bactericidal way from the MIC concentration. A substance is determined to be bactericidal when it has the ability to kill the bacterial cell, whereas bacteriostatic molecules only inhibit cell growth. Such data is relevant to provide information about the potency of action of the molecule. However, it is a valid concept under the pre-established and controlled conditions of experimentation, as it can vary according to the type of bacteria, amount of inoculum, drug concentration and duration of the test. These changes are therefore seen in clinical practice, where the conditions described are as variable as possible [18]. For this reason, it is necessary to combine this information with pharmacokinetic and pharmacodynamic data, to provide a more significant prediction of in vivo efficacy.

### 2.2. Myrtenol Time-Kill Kinetics against S. aureus

**Figure 1:**
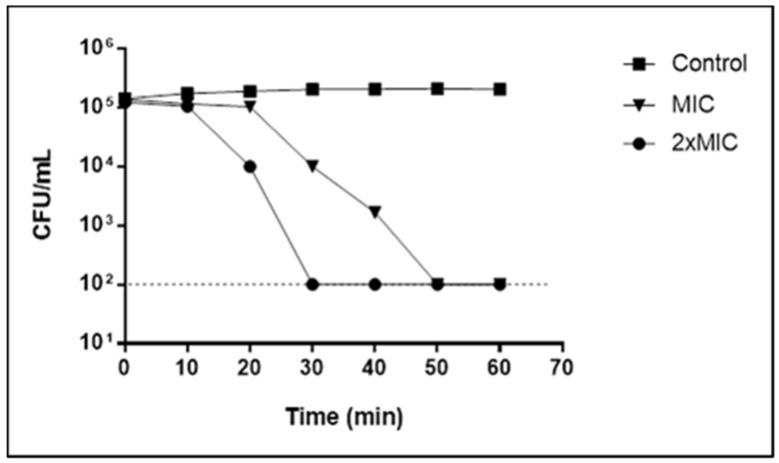
Time-kill curve for *Staphylococcus aureus* (LM-297) exposed to myrtenol at MIC and 2× MIC concentrations.

When investigating the action time of myrtenol against *S. aureus*, it is observed that after 50 min in the concentration equivalent to MIC, there is no more detection of viable cells. This time is reduced to 30 min when they are exposed to myrtenol in 2× MIC (Figure 1).

These data reinforce the results obtained in the MBC determination, whose values show a bactericidal effect of myrtenol from the MIC concentration. Although MIC and 2× MIC are bactericidal, the time of bacterial death is reduced with increasing concentration, as seen in the time-kill curve (Figure 1). Thus, the higher the concentrations of these phytochemicals, the shorter the time required to obtain the bactericidal effect.

### 2.3. Molecular Docking Analysis

**Figure 2:**
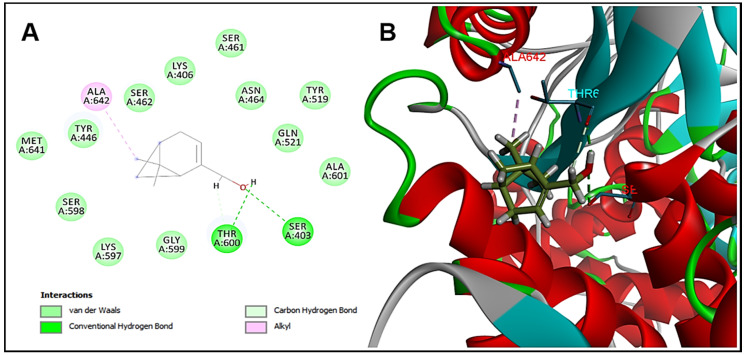
Molecular docking analysis. (**A**) Two-dimensional and (**B**) three-dimensional representation of myrtenol interactions in the PBP2 active site.

Molecular docking was performed in order to identify the possible target of myrtenol in the bacterial cell. The interaction of the substance with penicillin-binding protein 2 (PBP2) is observed, which is a transpeptidase that acts in the synthesis of the cell wall, being the target of some antimicrobial agents. For the validation of protocols performed in molecular docking, redocking was performed and the value of RMSD (root mean standard deviation) was used to analyze the accuracy of molecular docking. To be considered a valid docking, the RMSD values must remain in the range of 0–2 Å [19]. The RMSD value for the tested enzyme remained in the acceptable values (0.37 Å), Moldock Score −125.2 kcal/mol. Myrtenol showed binding energy −52.3 kcal/mol with PBP2 and, analyzing the interactions with the active site of the enzyme, the hydroxyl of myrtenol performed hydrogen bonding interactions with the residues of Ser403 and Thr600 and Van der Walls hydrophobic interactions with Lys406 (Figure 2). These interactions are necessary for effective anchoring at the active site of PBP2 and are carried out by β-lactam drugs [20].

The antibacterial mechanism of the action of myrtenol is not yet completely elucidated. In this work, the results indicate that PBP2 is a possible target for myrtenol to act against *S. aureus*. Thus, the substance would act by interfering in the synthesis of the bacterial cell wall, leading to cell death [19,20]. It is important to note, however, that these are preliminary results that help guide future in vitro and in vivo studies in order to clarify the exact mechanisms of action of myrtenol.

### 2.4. Association Test of Myrtenol with Antibacterial Drugs against S. aureus

Antibacterials are often used in combination, so it is relevant to understand the possible interactions between myrtenol and some commonly used drugs. Synergistic effects were found in the combination of myrtenol with gentamicin and additives in the association with ciprofloxacin, for all strains tested. The association of myrtenol with oxacillin, on the other hand, resulted in an indifferent effect. No antagonistic effects were observed with any drug tested (Table 2).

The combination of natural products and antimicrobials has been shown to be quite effective and promising for use in clinical practice. The use of associated substances makes it possible to reduce the required administration dose of each drug, while also reducing dose-dependent toxic effects. In addition, the association of natural products with antimicrobial agents can be an effective strategy to combat resistant strains. These phytoconstituents can work by several strategies, such as inhibition of target modifying and drug degrading enzymes or as efflux pumps inhibitors. Thus, they can act as bacterial resistance modifying agents, restoring the effectiveness of commercial antimicrobials or even have greater potency of action by acting in different mechanisms, achieving effectiveness against resistant strains [21,22].

The results indicate that myrtenol, despite having an indifferent effect when combined with oxacillin, in combination with gentamicin and ciprofloxacin increased the antimicrobial effect in a synergistic or additive way, which suggests the possibility of reducing the viability of the strains using a smaller concentration of these substances, and it is possible, consequently, also to reduce the side effects resulting from the administration of these drugs. It is interesting that association and modulation studies are carried out using strains resistant to multiple drugs, to verify whether the combination of myrtenol with other drugs is able to alter the resistance of these bacteria.

### 2.5. Antibiofilm Effect of Myrtenol against S. aureus

**Figure 3:**
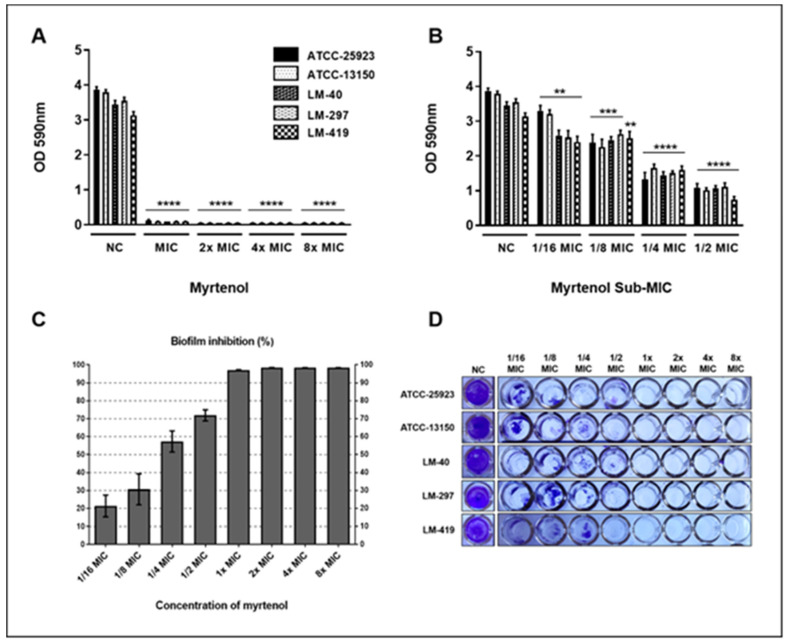
Myrtenol antibiofilm effect against *Staphylococcus aureus*. (**A**) Suprainhibitory concentrations; (**B**) Subinhibitory concentrations; (**C**) Percentage inhibition of biofilm formation; (**D**) View of biofilms formed in vitro by *S. aureus* in the presence and absence of myrtenol. NC: negative control. Statistical analysis compared to negative control: **** *p* ≤ 0.0001; *** *p* ≤ 0.001; ** *p* ≤ 0.01.

The antibiotic activity of myrtenol was evaluated based on the ability to inhibit the formation of *S. aureus* biofilms in vitro. Myrtenol showed a strong ability to inhibit the formation of the biofilm formed by *S. aureus*, in all tested concentrations. Significant differences were observed between the treated groups and the control group without treatment (Figure 3).

Myrtenol was able to strongly inhibit biofilm formation by all *S. aureus* strains used in this study from MIC (Figure 3A). In subinhibitory concentrations, myrtenol did not have a stimulating effect on the production of biofilm, being able to reduce the formation of biofilm, even in concentrations below the MIC (Figure 3B–D).

After 1/4 MIC, there was more than a 50% reduction in biofilm formation by *S. aureus* (Figure 3C,D). From the MIC, up to the maximum concentration used in this study (8× MIC), it is possible to observe that myrtenol promoted an inhibition greater than 90% of biofilm formation by *S. aureus*. Kwasny and Opperman [23] classify as good antibiofilm activity when a substance is capable of inhibiting ≥80% of biofilm growth and inhibit ≥40% of planktonic growth compared to untreated controls. Thus, it is observed that myrtenol has good antibiofilm activity from MIC onwards, in suprainhibitory concentrations, where there is ≥80% inhibition of biofilm growth and 100% inhibition of planktonic growth.

Similar results of myrtenol antibiofilm activity were found by Selvaraj et al. [14], whose data show that myrtenol attenuates MRSA biofilm considerably, in addition to inhibiting the production of major virulence factors of MRSA, such as lipase, hemolysin and staphyloxanthin. In addition, it affected the slime synthesis, autoaggregation, autolysis, and eDNA production in MRSA.

It has been observed that several classes of antibiotics, including β-lactams, at suboptimal concentrations, increase the potential for biofilm formation by different mechanisms. The induction of biofilm formation in subinhibitory concentrations is a clinically relevant process, because during the treatment of the infection, a part of the microorganism population is exposed to suboptimal concentrations, even when the recommended conditions of use of the drug are followed. Thus, low doses of these drugs can interfere with the course of the infection, complicating the treatment of these diseases [6,24]. However, myrtenol was able to reduce biofilm formation, even at concentrations below MIC, and potentially promising antibiofilm of the substance is evidenced, which should be further investigated and evaluated also against other microorganisms, in order to obtain a new clinical agent to combat this relevant virulence factor.

The ability of some microorganisms, such as *S. aureus*, to form biofilms, contributes to antibacterial resistance and therapeutic failures [25], so the development of effective tools to remove biofilms not only improves the treatment of biofilm-related infections, but can also potentially offer benefits to slow the spread of antibiotic resistance [26]. Since these results show that myrtenol is a potential antibiofilm agent, being able to act even in suboptimal concentrations, it is relevant that studies be further developed, evaluating its activity against biofilms formed by other species.

Since a substance can act by the most diverse mechanisms, such as interfering in key enzymes for biofilm formation, matrix-targeting enzymes, adhesion factors to the substrate, adhesion proteins involved in cell-cell aggregation or the biosynthesis of proteins important for biofilms formation and maturation [27], it is of great importance that the mechanism of action of myrtenol against biofilms is also elucidated, investigating its performance on possible molecular targets and using this information for future clinical applications.

### 2.6. In Silico Studies of Myrtenol Lipinski’s Parameters

The theoretical potential of myrtenol as a candidate for a new drug was assessed by the in silico parameters. According to the values expressed in Table 3, calculated by the online program SwissADME, it is possible to predict whether the myrtenol molecule may be a candidate for a drug, based on the rules of Lipinski [28], Ghose [29], Veber [30], Egan [31].

According to Table 3, the molar mass of myrtenol has a value of less than 500 g/mol, which meets the Lipinski criterion [28], where there should be no problem regarding the distribution aspect, as it is more easily transported than larger molecules. However, for Ghose parameters [29], the molecular mass must be between a range of 160–480 g/mol, so the molar mass of myrtenol, which is 152.23 g/mol, violates this rule. Log P is the partition coefficient of a molecule in n-octanol and water. To be considered an important parameter in the preparation of a candidate compound for a drug, Log P is related to the hydrophobicity of the molecule in the drug due to the ability to cross plasma membranes. However, molecules that are too hydrophobic tend to be more toxic, due to their ability to stay longer in the body. According to Table 1, the value of Log P was 2.40 and met the standards of the Lipinski rules (Log P_o/w_ ≤ 5) [28], Ghose (Log P_o/w_ ≤ 5.6) [27] and Egan (Log P_o/w_ ≤ 5.8) [31].

The hydrogen acceptor and donor values shown in Table 3 met the parameters of the rule of 5, where the number of hydrogen acceptors must be ≤10 and hydrogen donors must be ≤5. According to all the parameters presented by the Lipinski rule, myrtenol presents an excellent theoretical oral bioavailability. However, according to Veber, molecules that have TPSA values ≤140 Å and the number of rotatable connections ≤10 have a high probability of oral availability. In this way, myrtenol can display a high prospect of being employed orally. Solubility is an important feature for the absorption and distribution of the molecule in the body. Having a soluble compound favors medication planning, especially in formulation and manipulation. The Log S (coefficient of solubility determined by the Ali method [32]) of myrtenol presented a value of −3.32, indicating that the compound is soluble according to the class shown in Table 1.

Based on the above, in silico results show a favorable pharmacokinetic profile for this substance. Myrtenol has characteristics that suggest a good drug candidate and can display a high prospect of being used orally, with excellent theoretical oral bioavailability and good solubility, which can guarantee adequate absorption and distribution in vivo.

## 3. Materials and Methods

### 3.1. Substances

The myrtenol and the antibacterials gentamicin, ciprofloxacin and oxacillin were obtained from Merck/Sigma-Aldrich^®^ (Darmstadt/Germany). These substances were properly weighed and solubilized in dimethyl sulfoxide (DMSO) at 5% and Tween-80 at 2%, to obtain emulsions in the concentrations necessary for use in the tests.

### 3.2. Strains

In this work, strains of *Staphylococcus aureus* from clinical isolates were used, which belong to the MICOTECA of the Antibacterial and Antifungal Activity Research Laboratory of the Federal University of Paraíba, Brazil: LM-02, LM-40, LM-45, LM-182, LM-232, LM-297, LM-314, LM-418, LM-419, LM-443. In addition, the American Type Culture Collection strains ATCC-25923 and ATCC-13150 were used as controls. For use in the assays, bacterial suspensions were prepared in 0.9% saline solution, from fresh cultures, and adjusted to the McFarland standard 0.5 scale.

### 3.3. Minimum Inhibitory Concentration (MIC) and Minimum Bactericidal Concentration (MBC)

The MIC determination was performed based on the standard recommendations [33], using the broth microdilution technique in a 96-well plate, to obtain different concentrations of myrtenol. At the same time, the sterility controls of the culture medium, viability of the strains and interference of the vehicles used in the preparation of myrtenol emulsions (DMSO and Tween-80) were also performed. MIC is defined as the lowest concentration capable of causing the complete inhibition of bacterial growth after 24 h at 35 ± 2 °C.

After MIC reading, MBC was determined by removing aliquots from the microdilution plates in the wells corresponding to concentrations equivalent to MIC, 2× MIC, 4× MIC and 8× MIC and inoculating in new plates containing only culture broth. All controls were performed in parallel. MBC is defined as the lowest concentration capable of causing the complete inhibition of bacterial growth after 24 h at 35 ± 2 °C [17,34].

### 3.4. Time-Kill Kinetics

The determination of the action time of myrtenol against *S. aureus* (LM-297) was carried out, exposing the microorganisms to concentrations equivalent to MIC and 2× MIC during times 0, 10, 20, 30, 40, 50 and 60 min. After incubation on Mueller Hinton agar, bacterial colonies were counted. Bactericidal activity was defined as a ≥3-log reduction in bacterial counts (log_10_ CFU/mL) [35].

### 3.5. Molecular Docking

The chemical structure of myrtenol had its geometry optimized using the program Hyperchem (v. 8.0.6/Hypercube^®^/Florida/EUA), using the molecular mechanics method (MM+) and the semi-empirical method AM1 (Austin Model 1). The enzyme was obtained from Protein Data Bank (www.rcsb.org), together with its co-crystallized inhibitor, presenting the code 1MWT (2.2 Å) [20]. Molecular docking was performed using the Molegro Virtual Docker (MVD) software (v. 6.0.1, Molegro ApS^®^/Aarhus/Denmark), using the standard parameters of the software, removing the water molecules and generating a template in the co-crystallized inhibitor of the PDB enzyme.

### 3.6. Association Test

To check the effect of the association of myrtenol with the antibacterials gentamicin, ciprofloxacin and oxacillin, the checkerboard method was performed. Thus, different concentrations of myrtenol (8× MIC, 4× MIC, 2× MIC, MIC, 1/2 MIC, 1/4 MIC and 1/8 MIC) were combined with different concentrations of antibacterials (8× MIC, 4× MIC, 2× MIC, MIC, 1/2 MIC, 1/4 MIC and 1/8 MIC) and then microbial inoculum was added. All controls were performed in parallel. The reading of the experiment was done after incubation at 35 ± 2 °C for 24 h, to observe the presence or not of the visible bacterial growth. The effect produced between the combination of myrtenol and antibacterials was determined by the fractional inhibitory concentration index (FICI). This index was calculated by the sum of fractional inhibitory concentrations (FIC), where FIC_A_ = (MIC of substance A in combination)/(MIC of substance A alone) and FIC_B_ = (MIC of substance B in combination)/(MIC of substance B alone), thus FICI = FIC_A_ + FIC_B_. The association was defined as synergistic for FICI ≤ 0.5, as additive for 0.5 < FICI < 1, as indifferent for 1 ≤ FICI < 4, and as antagonistic for FICI ≥ 4 [34,36].

### 3.7. Antibiofilm Effect

To determine the antibiofilm potential of myrtenol, microdilution plates were incubated with brain heart infusion (BHI) broth, containing different concentrations of myrtenol and bacterial inoculum. The negative control was carried out containing only culture broth and inoculum. After 24 h incubation at 35 ± 2 °C, the contents of the wells were discarded and these were gently washed with sterile distilled water, in order to remove planktonic cells, reserving them for drying at room temperature. After drying, 1% violet crystal solutions were transferred and left to stand for 40 min. The dye was discarded and its excess in the tube walls were removed by washing with distilled water. Once dry, the tubes received absolute ethanol and, after 30 min at rest, the plate was read on a microplate spectrophotometer (Multiskan GO/Thermo Fisher Scientific Corporation^®^/Vantaa/Finland) at 590 nm [37]. All analyses were performed in quintuplicate. Statistical significance was determined by pair-wise testing using the t-test and the results were considered statistically significant when *p* < 0.05 for the rejection of the null hypothesis. For this plotting, GraphPad Prism (v. 6.0 for Windows/San Diego/California/EUA) software was used. The percentage of biofilm inhibition was calculated using the following formula: % of biofilm inhibition = [(OD_590_ control − OD_590_ test)/OD_590_ control] × 100.

### 3.8. In Silico Studies of Lipinski’s Parameters

Violations of the rules of Lipinski [28], Ghose [29], Veber [30], Egan [31] help to evaluate the pharmacokinetic characteristics of drug candidate substances. The following parameters were evaluated about myrtenol: physicochemical properties, lipophilicity, water solubility and druglikeness, using the free online software SwissADME (www.swissadme.ch) (Swiss Institute of Bioinformatics^®^/Lausanne/Switzerland). Such results were analyzed using the rules of Lipinski [28], Ghose [29], Veber [30], Egan [31].

## 4. Conclusions

Based on the above, this study showed that myrtenol has antibacterial activity against *S. aureus*, as a bactericidal agent that probably acts by interfering with the synthesis of the bacterial cell wall, with PBP2 as one of the targets. The effects of the combination with antibacterials demonstrate favorable results for use in combination in clinical practice. Myrtenol has strong antibiofilm activity and is able to inhibit its formation, even at subinhibitory concentrations. The in silico results show a pharmacokinetic profile with good theoretical oral bioavailability and good solubility, indicating adequate absorption and distribution of the molecule in vivo. Thus, myrtenol has characteristics that suggest that it is a good drug candidate to treat infections by *S. aureus*, whether isolated or associated with other drugs, in addition to being able to combat its associated biofilms.

## Figures and Tables

**Table 1:** Minimal Inhibitory Concentration (MIC), Minimal Bactericidal Concentration (MBC) and classification of the effect of myrtenol against *S. aureus* strains.

*S. aureus*	Myrtenol			
	MIC	MBC	MIC:MBC	Effect
ATCC-25923	128 µg/mL	128 µg/mL	1:1	Bactericidal
ATCC-13150	128 µg/mL	128 µg/mL	1:1	Bactericidal
LM-02	128 µg/mL	128 µg/mL	1:1	Bactericidal
LM-40	128 µg/mL	128 µg/mL	1:1	Bactericidal
LM-45	128 µg/mL	128 µg/mL	1:1	Bactericidal
LM-182	128 µg/mL	128 µg/mL	1:1	Bactericidal
LM-232	128 µg/mL	128 µg/mL	1:1	Bactericidal
LM-297	128 µg/mL	128 µg/mL	1:1	Bactericidal
LM-314	128 µg/mL	128 µg/mL	1:1	Bactericidal
LM-418	128 µg/mL	128 µg/mL	1:1	Bactericidal
LM-419	128 µg/mL	128 µg/mL	1:1	Bactericidal
LM-443	128 µg/mL	128 µg/mL	1:1	Bactericidal

**Table 2:** Myrtenol in combination with different antibacterial drugs (checkerboard method).

Strains and Drugs	FIC	FICI	Effect ^1^
ATCC-25923			
Myrtenol	0.25	0.50	Synergism
Gentamycin	0.25		
Myrtenol	0.06	0.56	Additivity
Ciprofloxacin	0.50		
Myrtenol	0.06	1.06	Indifference
Oxacillin	1.00		
ATCC-13150			
Myrtenol	0.25	0.50	Synergism
Gentamycin	0.25		
Myrtenol	0.06	0.56	Additivity
Ciprofloxacin	0.50		
Myrtenol	0.06	1.06	Indifference
Oxacillin	1.00		
LM-40			
Myrtenol	0.25	0.50	Synergism
Gentamycin	0.25		
Myrtenol	0.25	0.75	Additivity
Ciprofloxacin	0.50		
Myrtenol	0.12	1.12	Indifference
Oxacillin	1.00		
LM-297			
Myrtenol	0.25	0.50	Synergism
Gentamycin	0.25		
Myrtenol	0.12	0.62	Additivity
Ciprofloxacin	0.50		
Myrtenol	0.06	1.06	Indifference
Oxacillin	1.00		
LM-419			
Myrtenol	0.25	0.50	Synergism
Gentamycin	0.25		
Myrtenol	0.25	0.75	Additivity
Ciprofloxacin	0.50		
Myrtenol	0.12	1.12	Indifference
Oxacillin	1.00		

^1^ Synergism: FICI ≤ 0.5, additivity: 0.5 < FICI < 1, indifference: 1 ≤ FICI < 4 and antagonism: FICI ≥ 4.

**Table 3:** In silico studies of Lipinski’s parameters of myrtenol.

Parameters	Myrtenol
Physicochemical Properties	
Formula	C_10_H_16_O
Molecular Weigth	152.23 g/mol
Num. Heavy atoms	11
Fraction Csp3	0.80
Num. Rotatable Bonds	1
Num. H-bonds acceptors	1
Num. H-bonds donors	1
Molar Refractivity	46.38
TPSA ^**1**^	20.23 Å^2^
Lipophilicity	
Consensus ^**2**^ Log P_o/w_^**3**^	2.40
Water Solubility	
Log S (Ali)	−3.32
Class ^**4**^	Soluble
Druglikeness	
Lipinski ^**5**^	Yes; 0 violation
Ghose ^**6**^	No; 1 violation: MW < 160
Veber ^**7**^	Yes; 0 violation
Egan ^**8**^	Yes; 0 violation
Bioavailability Score	0.55

**^1^** TPSA: Topological Polar Surface Area; **^2^** Consensus Log P_o/w_ = Average of all five predictions; **^3^** Log P_o/w_ = The partition coefficient between n-octanol/water; **^4^** Class = Ali class: insoluble < −10 < poor < −6 < moderately < −4 < soluble < −2 < very < 0 < highly; ^5^ Lipinski = MM ≤ 500; Log P_o/w_ ≤ 5; H-bond donors ≤ 5; H-bond acceptores ≤ 10; **^6^** Ghose = 180 ≤ MM ≤ 480; 20 ≤ No. of atoms ≤ 70; 40 ≤ Molar Refractivity ≤ 130; −0.4≤ Log P_o/w_ ≤ 5.6; **^7^** Veber = Num. Rotatable Bonds ≤ 10; TPSA ≤ 140 Å^2^; **^8^** Egan = Log P_o/w_ ≤ 5.88; TPSA ≤ 131.6 Å^2^.

## Abbreviations

ATCC	American Type Culture Collection
BHI	Cerebral Heart Infusion (Broth)
CFU	Colony Forming Units
DMSO	Dimethyl Sulfoxide
FIC	Fractional Inhibitory Concentration
FICI	Fractional Inhibitory Concentration Index
FIC	Fractional Inhibitory Concentration
MBC	Minimum Bactericidal Concentration
MIC	Minimum Inhibitory Concentration
MVD	Molegro Virtual Docker
MW	Molecular Weight
PDB	Protein Data Bank
RMSD	Root Mean Standard Deviation
TPSA	Topological Polar Surface Area
